# Response costs of mammography adherence: Iranian women’s perceptions

**DOI:** 10.15171/hpp.2016.15

**Published:** 2016-06-11

**Authors:** Mahsa Khodayarian, Seyed Saied Mazloomi-Mahmoodabad, Minoor Lamyian, Mohammad Ali Morowatisharifabad, Hossein Tavangar

**Affiliations:** ^1^Department of Health Education & Promotion, Shahid Sadoughi University of Medical Sciences (International Campus), Yazd, Iran; ^2^Department of Midwifery and Reproductive Health, Tarbiat Modares University, Tehran, Iran; ^3^Department of Nursing & Midwifery, Shahid Sadoughi University of Medical Sciences, Yazd, Iran

**Keywords:** Breast cancer prevention, Mammography adherence, Protection Motivation Theory, Response costs, Maladaptive coping modes, Directed content analysis

## Abstract

**Background:** Mammography as the most common secondary prevention method has known to be helpful in detecting breast cancer at the early stages. Low level of participation among women toward mammography uptake due to cultural beliefs is a great concern. This study aimed at exploring the perceptions of women about response costs of mammography adherence (MA) in Yazd, Iran.

**Methods: ** A qualitative study using semi-structured interviews was performed. Fourteen women,one oncology nurse, and a breast cancer survivor were purposefully interviewed. Interviews were transcribed verbatim and analyzed by directed content analysis method based on protection motivation theory (PMT).

**Results:** One main theme was emerged from the analysis namely called "response costs".Two main categories were also emerged from the data; (1) psychological barriers with six subcategories including "embarrassment," "worry about being diagnosed with cancer," "preoccupation with underlying disease," "misconception about mammography," "need for an accompanying person," and "internalizing the experiences of the others," and (2) maladaptive coping modes which encompassed three subcategories: "religious faith," "fatalism," and"avoidance and denial."

**Conclusion:** Useful information was provided about the response costs of mammography utilization based on the perceptions of women. Cognitive barriers may be decreased by conducting modifications in women’s awareness and attitude toward MA as well as changing the national health system infrastructures. Incorporating religious and cultural belief systems into MA educational programs through motivational messages is recommended.

## Introduction

Breast cancer, as a global health problem, is a prevalent cancer among women, worldwide.^1^ it is, also, the most frequently diagnosed cancer among women in countries of the Eastern Mediterranean Regional Office (EMRO) and the fifth common cause of death among women in Iran.

In general, fundamental changes in the socioeconomic status among developing countries have made a shift in the lifestyles of people in these countries to those of the industrialized countries. Such a shift in the lifestyle, which is associated with reproductive, hormonal, and dietary risk factors, has led to an increase in the burden of breast cancer.^2^

It is noteworthy that the age of onset for breast cancer among Iranian women is reported to be up to ten years earlier than that in the developed countries.^3^ Epidemiological studies based on the age-specific incidence in Yazd province showed breast cancer as the most prevalent cancer.^4^ Despite the annual increase in the incidence rate of the disease among Iranian women, there is a lack of recent evidence on its various aspects in Yazd, Iran.^5^

Mammography screening has been shown to be associated with early detection and, therefore, mortality and morbidity reduction of breast cancer.^6^ Although mammography screening programs are being implemented for all older than 40 women in several countries like the United Kingdom and the United States,^7^ there is no formal mammography screening program in Iran. Unfortunately, 70% of Iranian women with breast cancer are diagnosed in the advanced stages, when it is too late for successful treatment.^8,9^ It is estimated that patients with breast cancer living in the lower-income countries have lower 5-year survival rates in comparison with their counterparts in the higher-income countries at any stage of the diagnosis.^10^ Lack of mass media education and screening programs, poverty, poor access to health care facilities, cultural barriers, and poor breast awareness are associated with lower breast cancer survival in the developing countries.^11^

Based on those mentioned above and, also, the recommendation of the World Health Organization (WHO) on mammography as the only effective breast cancer screening method, it seems to be necessary to encourage Iranian women for mammography adherence (MA).^12^

There are some perceived barriers influencing the mammography behavior; for instance, Donnelly et al in a study on the Qatari women showed that socioeconomic factors and awareness of recommended national guidelines of breast cancer screening may influence breast health seeking behaviors.^13^ Moreover, Lwin reported the lack of mammography awareness among Singaporean women in a way that they did not believe on the necessity of mammography behavior.^14^ According to the findings noted by Thomas et al, Iranian women experience several barriers in relation with breast cancer prevention and control such as the lack of knowledge, personal functioning, motivation, availability of information, health communication, and support.^15^ In the present qualitative study, the framework for interview questions and data analysis was based on the protection motivation theory (PMT). PMT was originally proposed by Rogers in 1975. It is encompasses of two independent appraisal pathways resulting from fear appeals; threat appraisal and coping appraisal.^16^ people might respond adaptively or maladaptively to the health information.^17^ In the threat appraisal pathway, an increase in severity, vulnerability, and fear may result in a decrease in the probability of occurrence of the maladaptive response. In the coping appraisal pathway, people evaluate the usefulness of the behaviors (response efficacy) and their ability to perform, successfully, preventive behaviors (also called self-efficacy). Two important factors that might increase the likelihood of maladaptive response are intrinsic and extrinsic rewards (e.g., pleasure and social approval of unhealthy actions), and response costs (barriers). The key point in this theory is adaptive behavioral change, which is reinforced through motivation.^18^ Protection motivation is the result of these two cognitive pathways (i.e., intention to perform a recommended behavior like mammography).^19,20^ Because of the differences exist in cultural contexts, the perceptions of women in Yazd about the breast cancer preventive behaviors may be different from those of the other countries. Thus, the findings in the other cultures may not be applicable for Iranian women. This study was designed to explore the perceptions of women about response costs of MA and to provide more insight on this cultural sensitive topic.

## Methods

In this qualitative study, directed content analysis was employed to analyze the data obtained by in-depth interview approach. The semi-structured interviews were conducted after designing a flexible framework for interview questions based on the PMT. The method section was provided based on the consolidated criteria for reporting qualitative research (COREQ) guideline. In the current study, the following key question was explored: What are the perceptions of women about the response costs of MA?

The study participants were 12 women, one oncology nurse, and a breast cancer survivor, all from Yazd, Iran, who were invited to participate in the study through purposive sampling. The eligibility criteria for the women were considered to be as the following: (*a*) having the age of 35 years or higher, (*b*) not suffering from mental disorders based on their records, and (*c*) the ability to communicate. The inclusion criteria for the nurse were as follow: (*a*) being with at least one year of nursing experience in an oncology unit, (*b*) having, at least, a bachelor degree in nursing, and (*c*) being female. Breast cancer survivor and oncology nurse were elected as critical cases in this study. The critical cases were considered to be interviewed hoping to address the relations that were being studied.^21^ Since oncology nurses have close interaction with breast cancer patients and their families, they were considered as key informants or experts in the study. Their perceptions and experiences were valuable to arrive a deep understanding of the effective factors on women’s breast health-seeking behaviors. Since the real experience of the participants is important in a qualitative study, a breast cancer survivor was chosen as a key informant, as well. The breast cancer survivors may have a good experience and deep understanding about the process of diagnosis and treatment of the disease. Moreover, as factors like nulliparity and the old age at the first live labor could enhance the chance of breast cancer occurrence, two old girls were interviewed as the persons at the higher risk for the disease. Thus, it was possible to get more insight about the subject. Maximum variation in sampling was considered with the participants’ age, marital status, education, occupation, and the breast disease history.

The interviews were continued until data saturation was appeared. In other words, emerging all possible aspects and domains of the main theme and categories was confirmed by the researchers. The pilot interviews were conducted with three women and the final form of the interview was prepared. The framework for the interview questions was the same in all three different groups of the participants (healthy women, oncology nurse, and breast cancer survivor). Meanwhile it was very flexible because of using semi-structured and in-depth individual interviews. So, when needed, some necessary changes were made in the interview questions by the interviewer. The first researcher was the main interviewer and data analyzer. A set of 14 audio-taped interviews was carried out from September to December 2014. The other researchers, who were faculty members affiliated with the Yazd University of Medical Sciences, Yazd-Iran and Tarbiat Modares University, Tehran-Iran, monitored and revised the data analysis process, step by step, to fulfill the systematic analysis. Fourteen participants aged 35 and older participated in the study. All were living in Yazd city, Iran. Table 1 presents further demographic data.

**Table 1:** Participants′ demographics (n = 14)

**Variables**	
Age, mean (SD)	39.42 (4.61)
Range	35-48
Marital status, n (%)	
Single	2 (14.3)
Married	10 (71.4)
Divorced	2 (14.3)
Education, n (%)	
Primary school	1 (7.1)
School diploma	6 (42.9)
Higher education	7 (50.0)
Occupation, n (%)	
Housewife	1 (7.1)
Employed	13 (92.9)
Breast disease history, n (%)	
Yes, Benign diseases	4 (28.6)
Yes, Breast cancer survived	1 (7.1)
No	9 (64.3)

The interviews were performed with prior appointment with the participants and they lasted for 30-90 minutes in a quiet and calm room at a clinic near to the participant’s home. The interviews started with open and general questions; however, the questions became gradually more detailed as the interviews were being progressed. The interviewer tried to keep the participants in the main path of the study in order to achieve the research objectives. The main focus of the interview questions were “what is the meaning of cancer in your idea?,” “what factors may prevent you from doing mammography?,” and “what are the ideas of the persons close to you about MA?”

The current study was an attempt to identify the response costs of MA among women in Yazd, as a part of a more comprehensive exploratory mixed method study for the first author’s doctoral dissertation. Directed qualitative content analysis based on the constructs of the PMT was used to data analysis, but, in this paper the results of the qualitative data analysis on the construct of response costs were, only, reported.

### Data analysis

In this study, qualitative directed content analysis was performed using Burnard’s 14-stage approach.^22^ Each interview was transcribed verbatim. The texts were imported into MAXQDA software version 10 for computer-assisted qualitative data analysis. The researcher read the texts several times to obtain a whole sense on the data. Unit of analysis and meaning units were considered. Open coding technique was adopted. The initial codes were organized into categories and subcategories. Different methods have been used in order to increase the trustworthiness of data analysis. For example, sufficient time was allocated for data collection and analysis. Constant comparative analysis was used throughout the process of analysis. The researcher bracketed her thoughts, preconceptions, and beliefs before and while performing the data analysis to ensure credibility. Some other strategies such as prolonged engagement with participants, faculty member’s revisions, and member checks were used to enhance the rigority of the data.

## Results

Analysis of the understandings of the Iranian women about their perceived response costs of MA led to formation of one main theme, two categories, nine subcategories, and 143 initial codes. Response costs factor was considered as the main theme. Psychological barriers and maladaptive coping modes were determined as categories. The subcategories underlying the category of psychological barriers were *embarrassment, worry about being diagnosed with cancer, preoccupation with underlying diseases, misconceptions about mammography, need for an accompanying person, and internalizing the experiences of the others*. The subcategories underlying the category of maladaptive coping modes were *religious faith, fatalism,* and* avoidance and denial*. Brief overview of the thematic structure of the results could be found in Table 2. The above mentioned categories and their related subcategories were explained separately in the following section.

**Table 2:** Thematic structure

**Main Theme**	**Category**	**Subcategory**	**Extracted Codes**
Response costs	Psychological barriers	Embarrassment	Embarrassment of older unmarried girls because of: 1) Narrow territory of to seek mammography 2) Late referral to a gynecologist 3) Being ashamed of disease follow-up 4) Bad impression of others about reasons to go to a gynecologist 5) Not being comfortable to announce the gynecologist visit time to the family
		Worry about being diagnosed with cancer	1) Worry about mammography result 2) Worry about hearing bad news
		Preoccupation with underlying diseases	Suffering from another physical problem
		Misconceptions about mammography	1) Harm of mammography for breast tissue 2) Uncertainty about the harmfulness of mammography rays
	Maladaptive coping modes	Need for an accompanying person	1) Loosing time to find an accompany
		Internalization the experiences of the others	1) Internalization unpleasant experiences about mammography 2) Easy access to unaware and close person to ask questions rather than health care providers
		Religious faith	1) Lack of belief in the possibility of being affected by breast cancer 2) Considering breast cancer as a gift of God or God's will 3) turning to spirituality, praying, and putting the problem in God's hand for cancer treatment 4) breast cancer: a type of punishment from the God because of errors in the past
		Fatalism	1) chance: a determinant factor 2) Having the feeling of lack of control or powerlessness against death
		Avoidance and denial	1) Unreal optimistism and given immunity as restrictive factors 2) Try not to think about breast cancer

### Psychological barriers

Embarrassment

Some participants stated that they were ashamed of mammography screening. In this regard, the old girls with no partner had comments different from the married women. They reported to experience a higher level of embarrassment than the married women because of their narrow territory to seek mammography, late referral to a gynecologist, being ashamed of disease follow-up, misunderstanding of the others about the reasons forgoing to a gynecologist, and, finally, not being comfortable to announce the family about the gynecologist visit. For instance, some participants reflected these concerns as follows:

“*In Yazd, when a girl goes to a gynecologist clinic, the others have a reproachful glareat her and think on what has happened that she visits a gynecologist. The family also has the same opinion*”, (P8). Another participant told: “*It is mentally difficult for a girl to announce her family about visiting a gynecologist. One can easily speak about visiting a dentist, but the case for a gynecologist appointment is not so easy*” (P7).

*Worry about being diagnosed with cancer*

Worry about the mammography result is another barrier. In other words, worry about hearing bad news (being diagnosed with cancer) and have no preparedness to confront with breast cancer may reduce the women’s motivation for MA. For example, one of the participants said:

“*If now I undergo mammography, the result may be OK. But I am afraid that if six months later I did the procedure, the result would be bad. Then, how should I deal with the disease. We always are waiting to hear good news. Nevertheless, I expect to be affected by breast cancer, as the disease has been occurred in my family*” (P9).

Preoccupation with underlying diseases

Women announced that suffering from another physical health problem may be a competing priority with breast cancer preventive behavior that may lead to a negative attitude toward mammography. For instance, one of the participants with diabetes said:

*“Since I have diabetes, I do not like to do anything with my physical health. I am mostly dealing with my blood sugar. Two years ago, I visited a doctor who prescribed me a medication that disturbed the level of my blood sugar. I am afraid that I visit a doctor for the breast issues, and then I had to take some medications which may increase the level of my blood sugar”* (P2).

Misconceptions about mammography

The most of the women participated in the study believed that mammography might be harmful for breast tissue. Some of them noted that breast cysts could be ruptured during mammography procedure. For example, one participant reflected her concern as it follows: “*Since I have many breast cysts and I have undergone a surgery once, I am worry about the stimulation and rupture of the cysts during mammography procedure. Won’t they [the cysts] be pressured a lot while mammography procedure?*” (P6). Uncertainty about the harmfulness of the mammography rays was another concern mentioned by women: “*I don’t know what kind of radiations mammography has? However, I have heard that it may be harmful for the body and should not be performed frequently*” (P13).

Need for an accompanying person

In the current study, the participants expressed that they need for a reliable and close person to accompany them while going to the mammography center. Because of this, they might lose the time or quite forget it. One of the participants said: “*I would like to be accompanied with a close person when going to mammography. When I find someone to be accompanied with, a long period of time has been passed*” (P5).

*Internalizing the experiences of the others*

According to the women’s statements, they could be highly influenced by each other. In other words, when women talk about mammography, they may describe their unpleasant experiences about it and thus decide not to uptake the mammography test:

“*I am a hairdresser. Once, one of my customers came to me just after undergoing mammography. She was very pale and had nausea. It seems that she was compressed. For that reason, I never want to undergo mammography*” (P6).

Also in relation to breast cancer, the women may ask their questions from unaware and close persons rather than health care providers because of having easy access to them. One participant explained this matter as follows: “*Everyone asks an intimate friend; one who understands her and is available. It is not easy for me to visit a doctor and consult her*” (P8).

### Maladaptive coping modes

*Religious faith*

Participants’ religious beliefs have been found to be strong barriers for seeking mammography. the belief in the possibility of not being affected by breast cancer and considering it as a gift of God or God’s will were other issues discussed by the participants. This consideration has been well expressed by one of the participants:

“*It is natural for people to believe in not being affected by breast cancer. Maybe God has created us in this way. If it was in another way, perhaps we were tortured. God may have put it inside us*” (P10).

Another woman said: “*It is God’s will that people are being affected by breast cancer. God draws some of us for the disease. God is the one who draws*” (P7).

Participants mentioned that the best way for dealing with cancer is turning to spirituality, praying, and delegating the problem to God. For instance, one of the participants commented as follows:

“*If I were diagnosed with breast cancer, I would pray for God more and more as I believe that the doctors cannot do anything for me and only God can cure me*” (P10).

Another woman stated that having breast cancer maybe a type of punishment from God because of mistakes people do in the past. She, also, noted that: “*Being affected by cancer is related to our deeds, and when someone has cancer, God examines her or him”* (P3).

Fatalism

Women participated in the current study mainly emphasized on the role of chance as an important factor which may limit the women’s power to prevent breast cancer. One of the participants quoted: “*All these things occur by chance. I think that chance plays a very important role in every event*” (P3). Another participant expressed that the cause of death for each person is being determined, previously, and an individual is considered to have a limited control over his/her death: “*Everybody must die in a way; one in a car accident, another experiences stroke, and some others may be diagnosed with breast cancer. There must always be a cause for death*” (P4).

Avoidance and denial

Some of the participants declared that they never think about breast cancer. Issues related to this subcategory were optimistic bias and an unreal assumption of being safe which may be considered as restrictive factors for mammography uptake. For instance, a participant said:

“*You know, I think I will never be affected by breast cancer, as in a proverb it is said ‘human beings think that all other people will die except themselves’*” (P1).

Another participant reflected the fact as follows:

“*When I think about something, for example when I have flank pain and think about it, it seems that the pain aggravates. It is really in this way; thinking to pain worsens it. I try not to think about breast cancer and if it comes to my mind, I remove it*” (P11).

## Discussion

The findings of the current study demonstrated a wide range of factors including psychological barriers and maladaptive coping modes that may affect the women’s adherence to mammography. Some of the aspects are new and there is no evidence about in the literature. These new findings are valuable as they show the effect of cultural context on the breast health-seeking behaviors of the Iranian women.

In the present study, according to the participant’s statements, embarrassment associated with mammography was considered as a vital response cost. This is consistent with those found by Fleming et al.^23^ The new and unique aspect of this study was the comments of the old girls about the shyness of referring to a gynecologist clinic or seeking help for breast cancer prevention. To the best of our knowledge, no report is available in the literature in this regard. The interviewer (the first author) is from Yazd city, Iran and notes that the old girls especially in the older ages are not willing to present any abnormal sign of breast or genital organs because of their fear about the opinions of the people around. In some cases, they might lose some suitable opportunities for marriage in the future. Therefore, in such a cultural context, it is considered inappropriate for an old girl to go to a gynecologist; in the other hand, the family may become remarkably worried about. Unfortunately, the old girls in Yazd are dealing with this problem and there is no evidence to compare the aforementioned results and, so, further investigation is needed.

Moreover, worry about being diagnosed with cancer was mentioned as another psychological barrier by the participants, which is in line with the findings reported by Whitaker et al.^24^

Similar with those reported by Lamyian et al,^25^ another psychological barrier found in the present study was preoccupation with underlying diseases which was considered as a part of the competing concerns.

There were, also, some misconceptions about the mammography procedure emerged during the qualitative analysis in the present study. For example, there was a belief that mammography might have severe complications for the breast tissue and, so, some participants especially those who had the history of breast cysts avoided from mammography uptake. The participants declared that they were less likely to undergo mammography because they follow what they hear from the others. Therefore, the impact of subjective norms is recalled as an important factor in the process of personal decision-making.^26^ Also, satisfaction with medical care was found to be important especially among asymptomatic women who decide to uptake mammography.^27^ Another factor was being worry about x-rays as a major barrier for mammography, which is consistent with the results of Sadikoglu et al.^28^

According to the participants’ comments, it was difficult for them to go to mammography clinics alone and they prefer to be accompanied by a close person like a sister or a close friend. It was concluded that embarrassment and fear of being diagnosed with cancer may affect the women’s self-esteem in MA. This issue could certainly waste the golden time for the early detection of breast cancer. Therefore, proper communication among the health care providers and women referring to the mammography centers may enhance the mutual trust and peace of mind. Accordingly, Meguerditchian et al showed that communication skills could be principal predictors of MA.^29^

As all the study participants in the present study were Muslim, religious belief was another issue that was emphasized by the participants. This concept was titled as maladaptive coping modes and grouped into three broad sub-categories. In a previous study, Zarghami stated that religion and spirituality were as significant barriers for Iranian women to seek medical screening and care in early stages of breast cancer; nevertheless, the role of such religious factors on cancer screening behaviors among women is not well understood.^30^ Some of the participants in the present study believed that being affected by breast cancer might be a God’s gift. The others expressed that the disease could be a probe or punishment from God, as they may already have committed a sin or guilt in their lifetime. These findings are similar to the results of a qualitative study by Azaiza and Cohen.^31^ Moreover, Hatefnia et al showed that religious beliefs have significant association with mammography uptake among Iranian women.^32^ The study participants believed that medical treatment for breast cancer is useless and God could cure them, only. Mitchell et al declared that it is likely for delay to be happened in presenting a self-discovered breast lump among women with strong religious beliefs; especially belief in “religious intervention in place of treatment.” The researchers suggested the interaction between clinicians and clergies to correct wrong beliefs to enhance breast health-seeking behaviors.^33^

Fatalism was conceptualized as “passively denying personal control” and encompasses the dimensions such as personal perceived lack of control over external events, luck, predetermination of a disease, and belief of inevitable death by a serious disease.^34^ The participants in the present study claimed that human beings have no control on his/her death and cancer diagnosis will, certainly, result in death. Spurlock and Cullins pointed out that fatalism could negatively influence breast cancer screening behaviors.^35^ Also, the results of the study conducted by Donnelly et al showed that Arab women living in Qatar reported being diagnosed with cancer as a bad luck.^36^ It was concluded that women’s perceptions on cancer fatalism is a cultural issue and should be further investigated especially in countries such as Iran that holds culturally diverse population groups.

The most of the women in this study noted that they have never thought about the likelihood of being diagnosed with breast cancer and the role early detection in the disease survival. They always supposed to have immunity against the disease; thus, they feel no need to have a mammogram. This finding is consistent with those of reported by Hasson et al. who revealed that 16.5% of the participants reported no thinking about undergoing mammography.^37^

## Conclusion

The current results gave us a more detailed picture on how Iranian women perceive response costs of breast health-seeking behaviors. Although, women are responsible for their own health within the context of breast cancer prevention, governmental policy makers are, also, responsible for provision of women’s health care facilities. In Iran, despite the high incidence of breast cancer, mammography screening is not an integrated routine service in the health care system. It was concluded that women in Yazd perceive some cultural-based barriers for MA. It is necessary to identify the priorities and the existing gaps in the country through accomplishing interdisciplinary scientific researches and specific interventions. Beside the development of women’s health policy in Iran, it is suggested that religious and cultural belief systems be incorporated into the educational programs applying persuasion- based health monitoring approach. Health educators may have good opportunities to design theory-based educational interventions within which the belief systems are being incorporated with the hope to initiate adaptive coping reactions.

## Ethical approval

Research protocol was approved by the Research Council and Ethics Committee affiliated by Yazd Shahid Sadoughi University of Medical Sciences, Yazd, Iran. Voluntary participation, anonymity, confidentiality, and withdrawing from the study at any time without any penalty were some issues that were explained for the participants. All the interviews were recorded with a sound recorder after obtaining permission and signing an informed written consent form by the participants.

## Competing interests

The authors declare no conflict of interest.

## Authors’ contributions

MK was the main investigator and con­tributed to development of research protocol, imple­mentation of the research, and drafted the manuscript. SSMM supervised the study and scientific in­tegrity of data collection and revision of the manuscript. ML was advisor to the qualitative study, par­ticipated in the design of the study and interpretation of data and revising the manuscript. MAM and Ht were advisor to the study, par­ticipated in the interpretation of data and revising the manu­script. All authors have read and approved the final manuscript.

## Acknowledgements

This study was financially supported by Shahid Sadoughi University of Medical Sciences, Yazd-Iran. The authors are grateful to all women who participated in the study.
